# Supplementary material for: Patterns of Intron Gain and Loss in Fungi
Source: PLoS Biol. 2004 Nov 30;2(12):e422. doi: 10.1371/journal.pbio.0020422 (PMC532390; doi:10.1371/journal.pbio.0020422)
Supplement: Table S1 — Also available at http://genes.mit.edu/NielsenEtAl/. (4.3 MB ZIP). [file pbio.0020422.st001.zip › NielsenEtAl/html/859.html]

AN8181.1.NCU09541.1.MG09524.1.FG07005.1


```
 CLUSTAL W (1.82) Multiple Sequence Alignments - Introns Inserted


Sequence 1: NCU09541.1	437 aa
Sequence 2: FG07005.1	391 aa
Sequence 3: MG09524.1	538 aa
Sequence 4: AN8181.1	475 aa
Alignment Length: 642 aa
Number Identitical Residues: 116 aa
Alignment Score (without introns) 6063


MG09524.1 	MSGPIFSNPSLLGFRQVGSSKCTDSSTLY0LYGARKSSCTIVISKLAVVRQGSPTRVHIT
NCU09541.1	-----------------------------~------------------------------
FG07005.1 	-----------------------------~------------------------------
AN8181.1  	-----------------------------~------------------------------
          	                                                            

MG09524.1 	CVTARMVSNPYLGSIHNTKPIKSVDQCPSARY0SYLNHSFSKSRMNAQVSFL0DGELSLI
NCU09541.1	--------------------------------~-----------MNAQITFL0DGTLRLI
FG07005.1 	--------------------------------~-----------MNAQVSFL0DGTYTLI
AN8181.1  	--------------------------------~----MENSLSLISAQVQFM0EDRLALV
          	                                      . : : :.**: *: :.   *:

MG09524.1 	HIPLNLYPTLLQPILQILLPQKESILQPEDEQGNGSPVASNSSRSWSQLGPHDQRPFLNV
NCU09541.1	HIPLPLYSTFLQPILQILLPP---------SQPLPS-ATPSP--PVGNFHHQDETTFLNI
FG07005.1 	HIPLSLYSSLLQPILRVLLPQ---------SQGAGG-LRDSPEYELQGLTSDGQHGFLNI
AN8181.1  	HIPLDLYPFFLNPILQVLFHEV--------APINEIRTETRGCDSPVEVSRHVQPAFLNI
          	**** **. :*:***::*:                             .  . :  ***:

MG09524.1 	SITPIECSVVCQTTWATSVFEPALQKLQQA---ARGKVSISKESYVALCVISAGMDAGSR
NCU09541.1	SITPLECSVVCPSSWARAVFEPAIARLPED---QAKTISVSKDAYAVFSVISAGMDAGSR
FG07005.1 	SVTPLECSVVCHTSWAQNVFEPVLKTLPRD---VAKIVSVSKESYMVLSVISAGLDAGGR
AN8181.1  	SITPVECSIMCPRQLADEYFAPLLERFSQLGSSSQSRLSISNDDFIAMQVYGEGLEAGQR
          	*:**:***::*    *   * * :  : . .::    :*:*:: : .: * . *::** *

MG09524.1 	VVDLSSPLALAGI~SIFFITTYYSDFILVPSKDKKNVVQALLARG-FEFTDNG--DSSSM
NCU09541.1	VVDLTCPLALAGI~PIFFITTYYSDFILVPTKDRRAVVETLRAKGGFVFVEEEGGDENGF
FG07005.1 	VMELTSPLALAGI~PIFFITTYYSDFILVPTKERENVVKSLIAKG-FELSDNE-------
AN8181.1  	VLELTTPLAMAGI2SIFFISTYFADYIIVPLKSKAQVIEALENRG-FQFEIST-------
          	*::*: ***:*** .****:**::*:*:** *.:  *:::*  :* * :  .        

MG09524.1 	MSLPRSGLGSYLNSHANSGAGSTMVSRRGSYNEHDNATPDSPLPSNADELQERTFALLKK
NCU09541.1	DGYGSRRGSVTMASGMGYHYASASSGNGGLSGMLTPMMPTTPPPSDAGELQLKTFETLKR
FG07005.1 	--------SNFITS------AANSQKQGG--GLPASSPPHEGLPLNVAELQTRIFGTLKK
AN8181.1  	---------EAFINNNQPSLNSPFSPISPLSTSTLSSPPATPPPSSLNELQKRTFSSLRR
          	           : .       :                *    * .  *** : *  *::

MG09524.1 	QSVQATVDTSLRLVHCSGHETSSHRLDG------EISAASPTAHLNRPSLSRMNTGNG--
NCU09541.1	RNVVPFVEPGLRLVHCSGREKTSIHGGGGGEGGRGHGHSGGNGTGYGGEIDGDGSANGGA
FG07005.1 	RNVAPNLEEALELVQCSGRETSQLA----------------NFTHQRPSISRHATGNG--
AN8181.1  	NNIIPYVDTSLRLVQCAAHHRFTND-----------------------------------
          	..: . :: .*.**:*:.:.                                        

MG09524.1 	-FSNGHSHRTWVDTVDTRLYTCLVAALAT-QPKFLSVTLAQDDPPS-LLLDKALVPMFGE
NCU09541.1	GYYPRRRRNSWIDTVDTKLYTSIISALVS-QPRFLSVTLAQDDPPS-LLLDKALVDMFGD
FG07005.1 	------RRPSWADNVDTKLYTCIISALVS-QPRFMSVTLAQDDPPS-LLLDKNLLDIFGD
AN8181.1  	------------DSSIAILRDALTTALVVDRPRFLSLTLTAADPAASLLLEQRLLPRFSS
          	            *.  : *  .: :**. .:*:*:*:**:  **.::***:: *:  *..

MG09524.1 	S------------LIGEAGGELAPIFLDLASLPFEATGIVSGVAGRLAREMS--------
NCU09541.1	S------------LVGPTEGTLVPIFLDLVNLPFEATGIVSGVAGRLVKDMPM-------
FG07005.1 	S------------LVGDTDAYLIPIFLDLKSLPLEATGIVCGVAGILVQDPQI-------
AN8181.1  	DPTFPADDDDSSLLLGSKEDILFPIMLDLRKLPLEATGIVCGVASRLADATHDRSEDSLA
          	..: .:....:: *:*     * **:*** .**:******.***. *.      :..: :

MG09524.1 	-----------------------------------------------------------D
NCU09541.1	-----------------------------------------------------------A
FG07005.1 	-----------------------------------------------------------A
AN8181.1  	ASATLSIDSSSPTYERSAQYVDIPVSNGSRRASKPNKSHSPGRKTSPPTHRLQPDLDSSF
          	:::: : .:::.: . ::.  . . :..:  ::....: :.. .::..:   ... .:: 

MG09524.1 	EGPELSYLSTARAGAVILSREQSAKAIEILGPLLSGDT--------
NCU09541.1	ESVELSYLSTARAGAVILSCEQAVTALGILEPLLKSQG--------
FG07005.1 	ASSELSYLSTAQAGAVILSEEQAVRAMSILKPLLSDEI--------
AN8181.1  	DAVEISFLSTARAGTIIVGEHELHRAMDALEAESQEPEKGIEALEL
          	 . *:*:****:**::*:. .:   *:  * .  .   .. .: .
```
